# Face-to-face learning enhances the social transmission of information

**DOI:** 10.1371/journal.pone.0264250

**Published:** 2022-02-25

**Authors:** Ashley Ransom, Brian LaGrant, Anthony Spiteri, Tamar Kushnir, Adam K. Anderson, Eve De Rosa

**Affiliations:** 1 Department of Psychology, Cornell University, Ithaca, New York, United States of America; 2 Department of Psychology, Human Neuroscience Institute, Cornell University, Ithaca, New York, United States of America; University of Bologna, ITALY

## Abstract

Learning from others provides the foundation for culture and the advancement of knowledge. Learning a new visuospatial skill from others represents a specific challenge—overcoming differences in perspective so that we understand *what* someone is doing and *why* they are doing it. The “what” of visuospatial learning is thought to be easiest from a shared 0° first-person perspective and most difficult from a 180° third-person perspective. However, the visual disparity at 180° promotes face-to-face interaction, which may enhance learning by scaffolding social perspective taking, the “why” of visuospatial learning. We tested these potentially conflicting hypotheses in child and young adult learners. Thirty-six children (4–6 years) and 57 young adults (18–27 years) observed a live model open a puzzle box from a first-person (0°) or third-person (90° or 180°) perspective. The puzzle box had multiple solutions, only one of which was modelled, which allowed for the assessment of imitation and goal emulation. Participants had three attempts to open the puzzle box from the model’s perspective. While first-person (0°) observation increased imitation relative to a 180° third-person perspective, the 180° observers opened the puzzle box most readily (i.e., fastest). Although both age groups were excellent imitators and able to take the model’s perspective, adults were more faithful imitators, and children were more likely to innovate a new solution. A shared visual perspective increased imitation, but a shared mental perspective promoted goal achievement and the social transmission of innovation. "Perfection of means and confusion of goals—in my opinion—seem to characterize our age" Einstein (1973) pg 337, *Ideas and Opinions*

## Introduction

Learning from others is the foundation of culture and cumulative knowledge [1] but also represents a physical conundrum. Visuospatial learning requires us to “see” the world as another does, which necessitates a deviation from an egocentric perspective. In some of the earliest work on social learning, Thorndike [2] noted that learning involves the transformation of a model’s actions (allocentric) to fit the observer’s (egocentric) perspective, which utilizes visual perspective taking (VPT). Yet, observation is insufficient for learning. Social learning also requires an understanding of a model’s intentions and goals, which utilizes social perspective taking (SPT). Early in life, humans are discerning learners who utilize SPT to increase the efficiency of social learning [3, 4]. VPT allows an observer to see *what* a model is doing. SPT allows the observer to know *why* the model is doing it. In this way, VPT and SPT work in tandem to support learning. Beyond literally seeing the world through another’s eyes, successful learning requires that we understand another’s mental perspective. Here we examined the potential interplay between visual and social perspective taking when learning to solve a complex visuospatial problem with multiple solutions, and further, how they integrate and exploit initial observational learning into self-guided exploration [5].

When learning a new visuospatial skill, such as how to tie a knot or play an instrument, an observer embodies the model’s external experience of the world. This visual perspective taking consists of two levels that develop sequentially and provide complementary information [6–8]. Level 1 is the ability to discern whether another person can see an object and is fully developed by 2-years [7, 9]. Level 2 is the ability to discern how an object appears to another person (e.g., object orientation) and develops during the preschool years [6, 8]. Here we examined young adult and child learners to consider how Level 2 VPT impacts the social transmission of visuospatial knowledge across development.

In our daily lives, we routinely interact with others from multiple viewpoints, and these changes in viewpoint alter the difficulty of VPT. VPT utilizes mental rotation, which increases in difficulty as angle of rotation increases [10]. Accordingly, visuospatial learning should be most difficult from observational viewpoints that necessitate mental rotation. Even more than a first-person versus third-person distinction, the magnitude of disparity between model and observer is crucial during learning. Prior studies found that imitation of specific actions is faster when seen from a 0° viewpoint compared to a 180° viewpoint [11–13]. Thus, observational viewpoint becomes a critical structural constraint on learning, particularly when the objective is fidelity in copying specific actions. Examining a shared first-person perspective (0° viewpoint) versus third-person perspectives differing in angular disparity (90° and 180° viewpoints) affords an examination of the role of mental rotation in observational learning.

Beyond a model’s external experience of the world, learning also requires the observer to understand a model’s internal experience of the world. Understanding a model’s intentions [14] and goals [3] promotes social learning. Rather than imitating all actions, SPT helps the learner discern a model’s inner state and thus supports learning of goals. Even young children use SPT to imitate intentional but not accidental actions [4]. While VPT is more difficult from a 180° relative to a 90° viewpoint, and most difficult relative to a 0° viewpoint, a 180° viewpoint allows for face-to-face interaction, which may scaffold SPT. Face-to-face interaction improves communication [15, 16] encourages social affiliation [15] and heightens visibility of the mouth and eyes, which facilitate the comprehension of mental states [17, 18]. In a learning context, face-to-face interaction may bolster attention to social cues that support learning such as eye contact, eye gaze, and smiling [19–21]. If SPT plays an important role in the social transmission of complex visuospatial knowledge, there may be a nonlinear relationship between model-observer viewpoint disparity and learning. Viewpoint disparity that enhances SPT (i.e., a 180° viewpoint) may actually facilitate the social transfer of knowledge.

Past studies on observational viewpoint and visuospatial learning relied on asocial models [12, 13]. In these studies, participants watched a video of a model and then imitated the model’s motor actions (e.g., simple hand movements). Only the model’s hands were visible, and the model never interacted with participants. Although the research we present here was informed by this literature, we moved away from these paradigms by examining visuospatial learning from a live, highly visible model. Furthermore, more than strict imitation, we examined the social transmission of real world “know-how” towards solving a complex visuospatial problem, a puzzle box. Beyond mimicking a model’s actions, we were interested in how viewpoint influences achievement of a goal (solving the puzzle box). We hypothesized that the social affordances of face-to-face learning may be powerful enough to reverse the difficulties of visual perspective taking so that visuospatial learning becomes easiest at a 180° viewpoint, promoting goal accomplishment. As such, the difficulties of third-person learning will be mitigated by face-to-face observation, with a 180° viewpoint resulting in greater puzzle opening ability than a 90° observational viewpoint, and potentially rivaling a 0° first-person viewpoint.

We examined how face-to-face learning affects the social transmission of visuospatial information between an observer and a model. Based on studies of observational learning in non-human primates and young children [22], we employed a puzzle box. Puzzle boxes are complex visuospatial tasks that, although artificial, are ecologically relevant as they capture individual differences in the social transmission of learning [23]. We utilized a type of puzzle box known as an artificial fruit box because they are analogous to a fruit that one must learn to open. Artificial fruit boxes have been used to study social learning in humans and non-human primates [24, 25]. They have multiple solutions, but all involve a sequence of hierarchical actions. As such, the puzzle box affords joint examination of imitation of the model’s actions and exploration of new solutions discovered by the observer from their own experience.

We examined children’s and adults’ tendency towards strict imitation versus innovation, i.e., solving the puzzle box using a repertoire of the model’s actions in a new order. A potent individual difference is age, which reflects both the amount of formal education as well as experience with visual and social perspective taking. We examined 4 to 6-years-olds; children of this age have just begun formal education and have developed Level 2 VPT [6, 8], which is a prerequisite for performance on our social learning task. Additionally, children of this age have developed theory of mind, which supports SPT [26]. The young adults in our study were expected to be better at mental rotation [27, 28], had more years of formal education, and presumably had more advanced skills that support social learning than the children. As such, this afforded an examination and potential generalization of our results across both expert and novice learners.

Observers watched a live model open a puzzle box from a 0°, 90°, or 180° viewpoint before attempting to open the box from the model’s perspective at 0°. The model performed both necessary and causally unnecessary actions when opening the puzzle box. Using the model’s sequence of actions to open the puzzle box was an index of imitation. We added unnecessary actions as an additional measure of imitation fidelity. There is evidence from prior studies that both children and adults will overimitate, or “blindly” copy, actions that are causally irrelevant to task success [29]. Coding of these unnecessary “flourishes” that were causally unrelated to the solution was taken as a measure of overimitation. To index puzzle solving efficiency independent of success in opening the box, which would likely be at ceiling level for adults, we measured how long it took to open the puzzle box. To examine the maintenance of observational learning and its evolution over time, we measured participants’ puzzle box solutions across three trials following initial observation of the model.

Finally, as a novel measure of implicit perspective taking, we allowed participants to choose where to sit after the model demonstration. If participants in the 90° and 180° conditions took an egocentric rather than allocentric perspective, then they should choose their original seat over the model’s seat. This behavior would indicate a preference for emulation rather than imitation of the precise actions of the model.

## Method

### Participants

Ninety-three participants (young adults, *n* = 57; children, *n* = 36) were tested over the course of an academic semester. An additional eight participants were excluded because of experimenter error or study incompletion. The adult participants were university students (18 to 27 years; *M* = 19.91_years_, *SD* = 1.61) who received course credit for participation. The child participants were 4 to 6-year-olds (*M* = 4.75_years_, *SD* = 0.81) who participated at a science museum or preschool. Children received a small prize (stickers or toy) for their participation.

Adult participants provided written consent. Child participants gave oral assent, and a parent/guardian provided written consent. The study protocol was approved by the university institutional review board and met recognized ethical guidelines.

### Materials

Prior to the study, adults completed the Autism-Spectrum Quotient Test (AQ-Adult), which quantifies level of autistic traits in adults [30]. This was an exploratory covariate assessing potential individual differences in SPT. Difficulty with SPT is well-documented among individuals with Autism Spectrum Disorder (ASD) [31, 32], and deficit in imitation is a quintessential feature of ASD [33]. Parents completed the Autism-Spectrum Quotient Test: Children’s Version (AQ-Child) [34], which quantifies level of autistic traits in children. The AQ-Child items are identical to the AQ-Adult items.

### Apparatus

**Fig 1:**
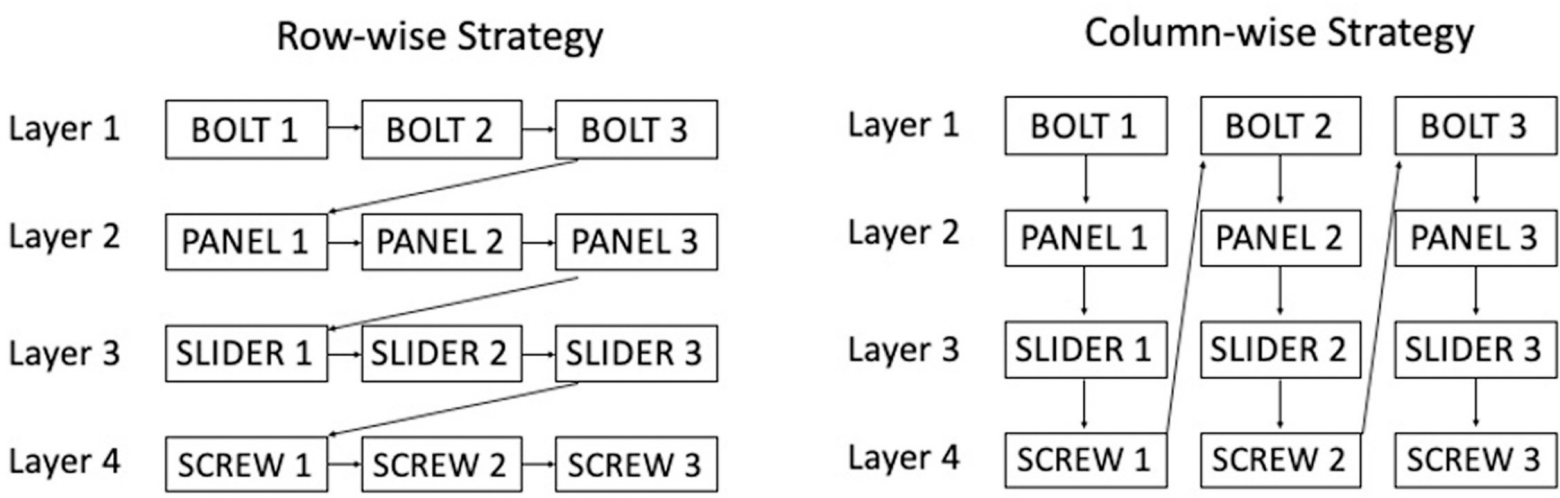
Two strategies for opening the puzzle box. The puzzle box consisted of four layers and could be opened using a row-wise (horizontal) or column-wise (vertical) strategy.

The social learning task utilized a colorful puzzle box similar to one used by Horner and Whiten [35]. The puzzle box hierarchy contained four layers with each subsequent layer accessed by opening the previous layer. Layer 1 was opened by removing three bolts. Layer 2 was opened by removing three panels. Layer 3 was opened by moving three sliders. Layer 4 was opened by using the bolts to turn three screws. The layers could be opened in a horizontal sequence— a row-wise strategy—or in a vertical sequence—a column-wise strategy (Fig 1).

### Procedure

Participants were tested individually in a quiet room.

#### Observation phase

**Fig 2:**
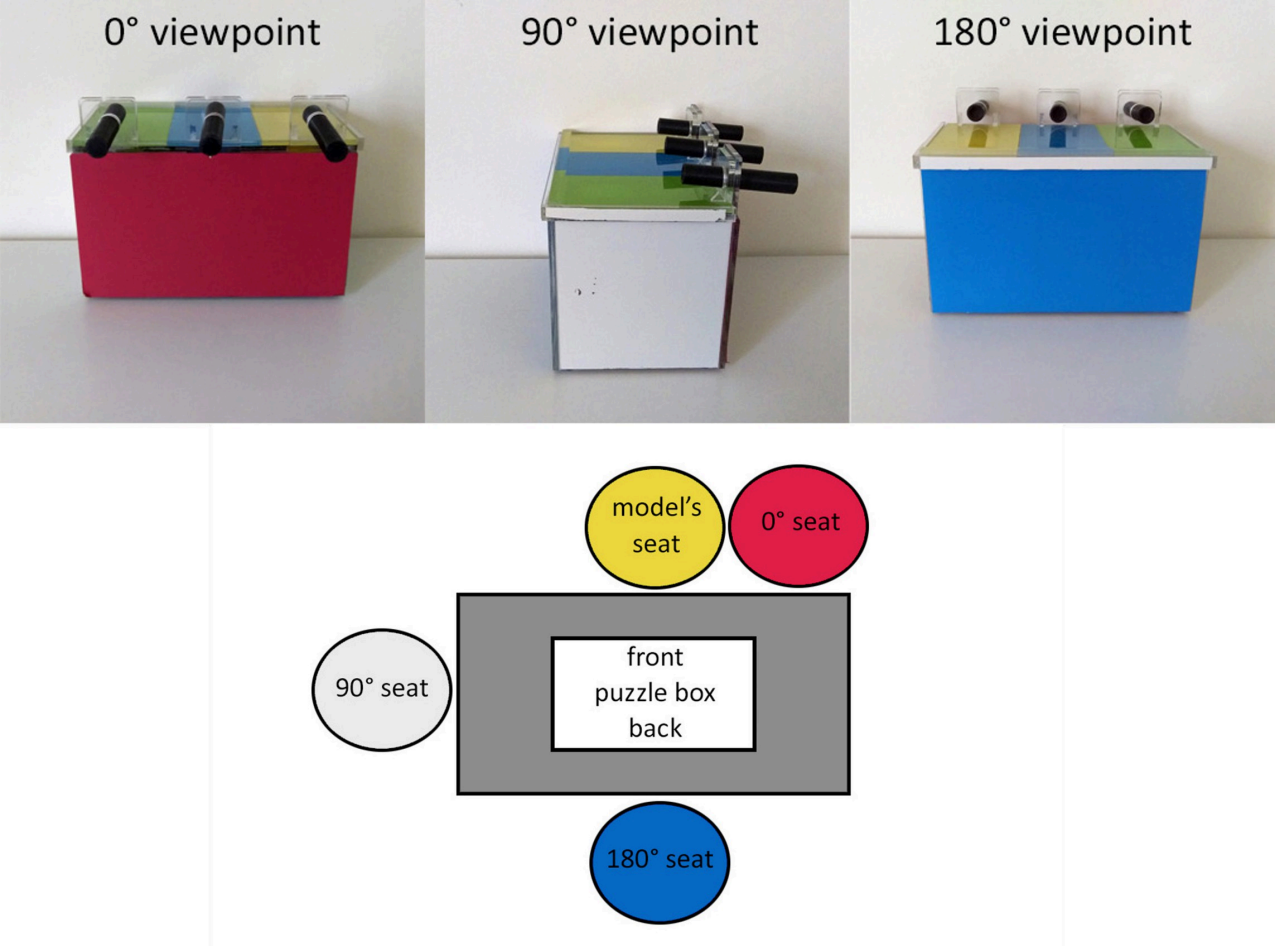
**a. (top) The Appearance of the Puzzle Box in Each Viewpoint Condition.** The puzzle box was opaque so that the inner machinery was invisible. The front of the puzzle box was red, and the back was blue, which allowed participants to differentiate the sides. **b. (bottom) The Testing Room Set-Up.** Participants in the 0° condition sat facing the puzzle box. Participants in the 90° condition sat facing the side of the puzzle box. Participants in the 180° condition sat facing the back of the puzzle box.

Participants were randomly assigned to watch a model open the puzzle box from a 0° viewpoint (*n* = 32; 20 adults, 12 children), a 90° viewpoint (*n* = 31; 19 adults, 12 children), or a 180° viewpoint (*n* = 30; 18 adults, 12 children) (Fig 2). We utilized four models during the course of the study. All were in their early twenties; two models were men and two were women. The puzzle box was placed on a table, and the model sat facing the front of the puzzle box. Participants in the 0° condition sat next to the model. Participants in the 90° condition sat perpendicular to the model. Participants in the 180° condition sat across from the model. The model’s actions were clearly visible from every viewpoint. The research sessions were video recorded from three angles corresponding to these three viewpoints (behind the participant, perpendicular to the participant, across from the participant) for later behavioral coding.

Participants were told to watch carefully because they would open the puzzle box later. The model opened the puzzle box by performing 12 necessary actions—each of the four layers was opened by performing three actions (Fig 1). The model opened the first three layers using only their hands, while the fourth layer required a switch to tool use (using bolts to turn screws). In addition to these 12 necessary actions, the model performed 14 causally unnecessary actions when opening the puzzle box (e.g., tapping a bolt against the box) so that we could measure overimitation (Table 1). The model opened the puzzle box with a row-wise strategy and moving from left to right. Adults watched the model demonstration once, and children watched the model demonstration twice.

**Table 1:** The 14 unnecessary actions performed by the model during the learning phase.

**Layer 1**	**Layer 2**
Stand Bolt 1 next to puzzle box	Place Panel 1 on table, facing self
Push Bolt 2 away from self	Place Panel 2 on top of Panel 1, facing left
Stand Bolt 2 next to puzzle box	Place Panel 3 on top of Panel 2, facing self
Stand Bolt 3 next to puzzle box	
**Layer 3**	**Layer 4**
Move Slider 1 down, up, down	Tap Bolt 1 on puzzle box three times
Move Slider 2 up, down, up	Return Bolt 1 to holder
Move Slider 3 down, up, down	Return Bolt 2 to holder
	Return Bolt 3 to holder

#### Pre-test seat choice

Once the model completed the demonstration, participants left the room while an experimenter prepared for the test trials. When participants re-entered, they were allowed to choose their seat upon returning to the test table. They could choose to sit facing the front of the puzzle box (0° viewpoint), the side of the puzzle box (90° viewpoint), or the back of the puzzle box (180° viewpoint). This served as a measure of viewpoint preference. If the participant did not choose the 0° viewpoint, then the puzzle box was turned so that it was facing the participant for the test trials. All participants were tested opening the puzzle box from the 0° viewpoint (i.e., the model’s perspective).

#### Test phase

Participants received three opportunities to open the puzzle box, which comprised three test trials. If a participant was unable to open the puzzle box, then an experimenter reset the puzzle box and initiated the next trial.

## Results

### Behavioral coding

From the video recordings, an experimenter, blind to condition, coded participants’ seat choice before beginning the test trials and the specific actions performed when opening the puzzle box on each trial. We used this coding to calculate four dependent variables—the number of layers opened as an index of accuracy, puzzle box solution type as an index of imitation of the model’s solution, time to opening the puzzle box as an index of learning, and the proportion of unnecessary actions performed as an index of overimitation. If a participant opened the puzzle box the way the model demonstrated (row-wise and left-to-right), then this was coded as the model’s solution. If a participant opened the puzzle box using a different method, then this was coded as a novel solution. We measured how long participants took to open the puzzle box in two ways. First, we measured time spent on each trial, which was defined as the number of seconds from touching the first piece of Layer 1 to the last piece of Layer 4. Second, as a more granular measure of intra-puzzle progress, we measured how long participants took to open each puzzle box layer, which was defined as the number of seconds from touching the first piece of the layer to the last piece of the layer. A second experimenter coded 20% of participants. Interrater reliability was high with intraclass correlations greater than .85 for each of the variables (layers opened, puzzle box solution, time by trial, time by layer, unnecessary actions).

### Individual differences in SPT

We calculated a percentage score for the AQ because some participants and parents did not answer all of the AQ items. Higher scores were used to index lower levels of SPT. Adult scores ranged from 20.67 to 57.33 with an average of 40.73 (*SD* = 8.72). Child scores ranged from 14.29 to 62 with an average of 37.16 (*SD* = 10.24). None of the participants had a score of clinical significance (a score of 64+).

A one-way ANOVA revealed no significant difference in AQ scores across viewpoint conditions, *p* = .48. As such, there was sufficient randomization of perspective taking traits across between-subject factors. Further, AQ scores did not account for a significant portion of variation in performance, all *p*’s > .05. Thus, it was not explored as a factor of interest but was controlled for in all analyses.

### Analysis plan

We examined various facets of performance on the puzzle box task including 1) proportion of layers opened (success), 2) proportion of layers opened using the model’s solution (imitation), 3) proportion of model’s unnecessary actions performed (overimitation), 4) time to open the puzzle box on each trial, 5) time to open each puzzle box layer. For each dependent variable, we conducted the same mixed model ANCOVA with trial (Trial 1, Trial 2, Trial 3) as a within-subjects variable, viewpoint (0° condition, 90° condition, 180° condition) during initial observation as a between-subjects variable, age group (adults, children) as a between-subjects variable, and SPT individual differences as a covariate. We tested for interactions between trial, viewpoint, age group, and SPT, and significant interactions were included in the model. We further examined time to open the puzzle box and unnecessary actions with a mixed model ANCOVA that included puzzle box layer (Layer 1, Layer 2, Layer 3, Layer 4) in place of trial. We included unnecessary actions as a covariate on the time analyses since performing these additional actions contributed to how quickly the box was opened.

Ten trials out of a total of 279 trials were excluded from the analysis because the experimenter did not reset the puzzle box completely (*n* = 2) or the participant chose to skip the trial (*n* = 8).

To examine viewpoint preference for opening the puzzle box, after initial observation of the model, we also examined whether participants chose the model’s seat, observer’s seat (their original seat), or novel seat before beginning the test trials. We did not include the 0° condition in this analysis because the model’s seat and observer’s seat were in the same orientation.

### Power analysis

We conducted a post hoc power analysis using the “simr” package in R, which calculates observed power through Monte Carlo simulations. We calculated observed power for the main effect of viewpoint on time to open the puzzle box because this index of learning was the focus of our study. The observed power for time to open the puzzle box across trials was 80.90% with an alpha level of .05.

### Puzzle box success

**Fig 3:**
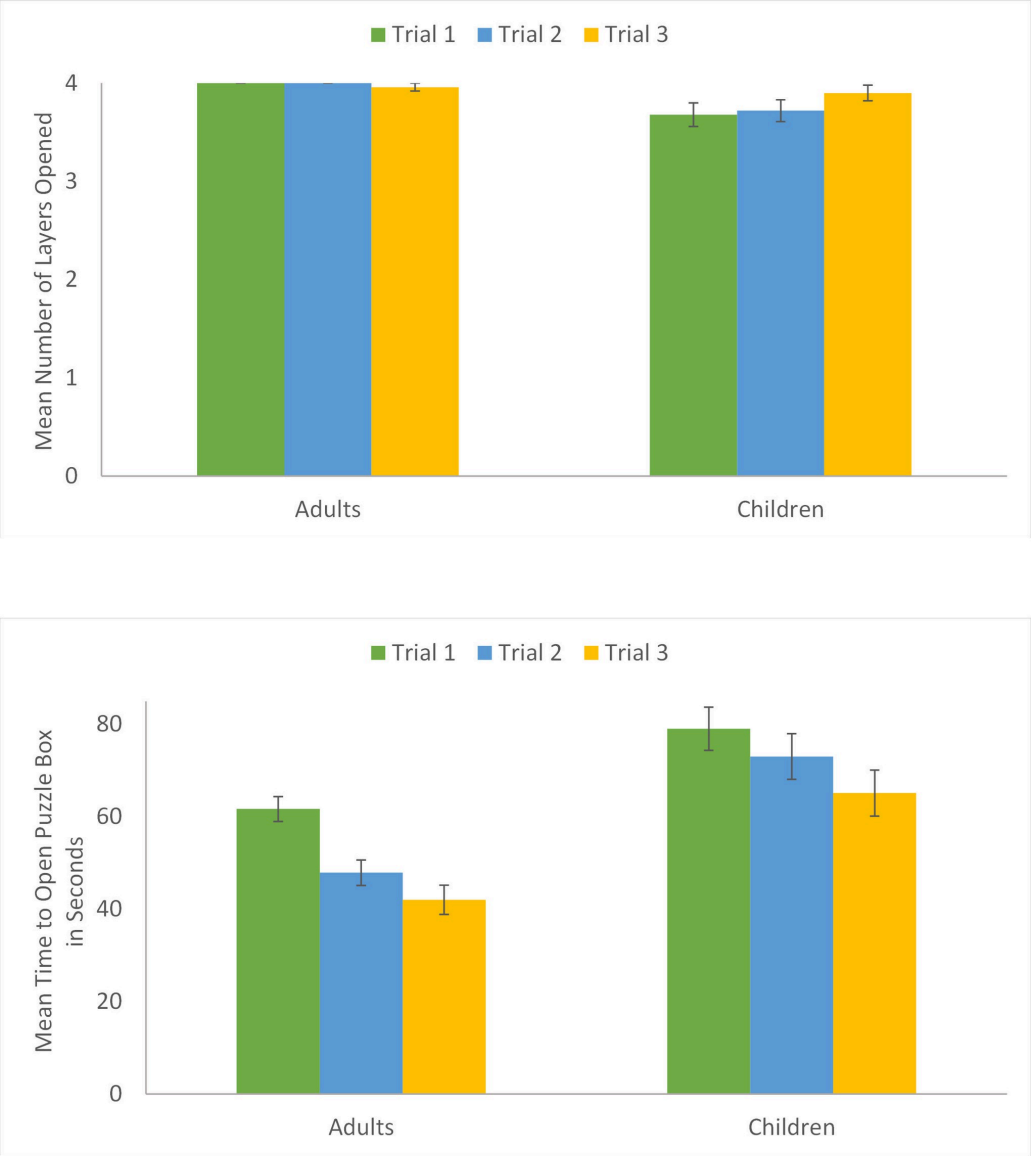
**a (top). Puzzle Box Success by Trial and Age Group.** Adults opened significantly more puzzle box layers than children. **b (bottom). Puzzle Box Time to Open Puzzle Box by Age Group.** Adults were significantly faster at opening the puzzle box than children.

Adults and children were successful at solving the puzzle box. As a measure of accuracy, we examined the number of puzzle box layers that participants successfully opened. There was a main effect of age group, *F*(1, 88.44) = 7.91, *p* = .006, η_p_^2^ = .08 (Fig 3A). Adults (*M* = 3.99 out of 4 layers, *SD* = 0.15) successfully opened more puzzle box layers than children (*M* = 3.76 out of 4 layers, *SD* = 0.58), 95% CI[0.07, 0.39]. There was no main effect of trial, *p* = .93, or viewpoint condition, *p* = .44. Adults were at near perfect performance on all trials, while child performance was best on the final trial.

### Puzzle box solution

As a measure of imitation, we examined the proportion of layers on which participants used the model’s solution when opening the puzzle box. There was a main effect of trial, *F*(2, 175.64) = 7.37, *p* < .001, η_p_^2^ = .08. Imitation of the model was greatest on the first attempt after observation and decreased with practice solving the puzzle box. Participants used the model’s solution more often on Trial 1 (*M* = .80, *SD* = .28) than on Trial 2 (*M* = .72, *SD* = .34), 95% CI[.02, .13], *p* = .007, or Trial 3 (*M* = .70, *SD* = .35), 95% CI[.05, .17], *p* < .001. There was no significant difference between Trial 2 and Trial 3, *p* = .29.

**Fig 4:**
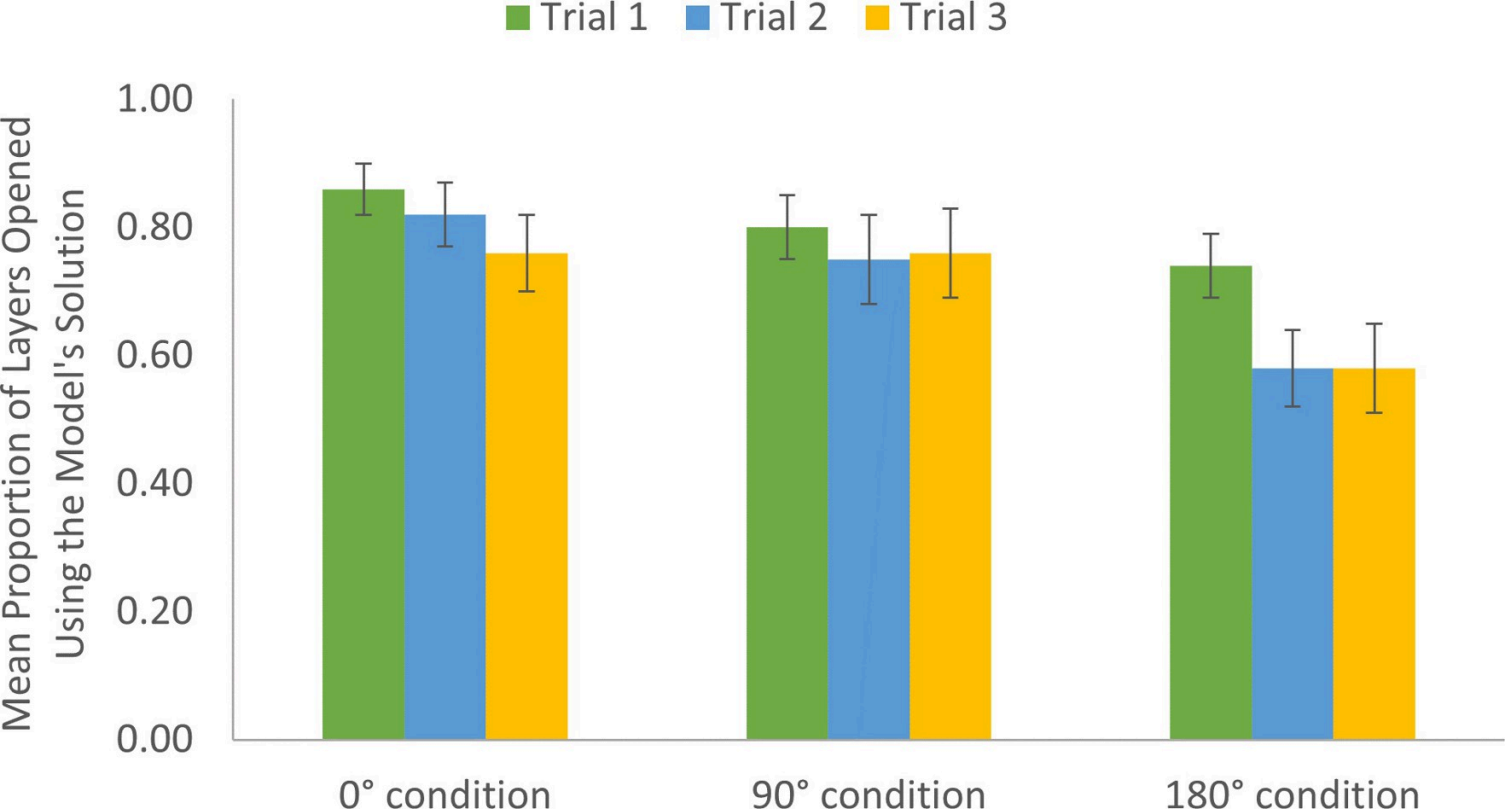
Use of the model’s solution by trial and viewpoint condition. Participants used the model’s solution significantly more on the first trial than on the second or third trials. The 180° condition used the model’s solution significantly less than the 0° condition and marginally less than the 90° condition.

There was a main effect of viewpoint, *F*(2, 87.83) = 4.04, *p* = .02, η_p_^2^ = .09 (Fig 4). Replicating prior results, imitation of actions was greatest when the participant shared the model’s perspective. The first-person 0° condition (*M* = .81, *SD* = .28) used the model’s solution more often than the 180° condition (*M* = .64, *SD* = .33), 95% CI[.05, .30], *p* = .007. The 90° condition (*M* = .77, SD = .34) used the model’s solution marginally more often than the 180° condition, 95% CI[.005, .24], *p* = .06. There was no significant difference between the 0° condition and the 90° condition, *p* = .39. More than a first-person versus third-person perspective, increased observer-model viewpoint disparity resulted in decreased imitation.

There was a main effect of age group, *F*(1, 89.05) = 31.40, *p* < .001, η_p_^2^ = .26. Adults (*M* = .85, *SD* = .28) were much more likely to imitate the model’s solution than children (*M* = .56, *SD* = .31), 95% CI[.19, .40], *p* < .001. Compared to adults, children were more likely to use a novel solution when solving the puzzle. When the observer shared the model’s vantage point during learning, this resulted in the greatest imitation of the model’s solution, while face-to-face learning resulted in the least imitation. Practice opening the puzzle box resulted in decreased imitation of the model’s solution.

#### Allocentric vs egocentric reference frame in imitation

As a more detailed analysis, we further explored participants’ solutions by examining the use of egocentric third-person (mirror reversed at 180°) or allocentric first-person motor actions (as observed at 0°), indexed by the direction in which they opened the puzzle box. The model demonstrated opening the puzzle box moving from left-to-right, which would appear mirror reversed, as right-to-left, for participants in the 180° condition. If these participants took the model’s visual perspective, then they should reverse their observed actions, opening the box from left-to-right. If, instead, they took an egocentric perspective, then they should open it from right-to-left.

Observers opened the majority of layers using allocentric model-based coordinates, moving from left-to-right (*M* = 73% of layers, *SD* = 29%). There was a main effect of viewpoint, *F*(2, 87.87) = 3.53, *p* = .03, η_p_^2^ = .07. Participants in the 180° condition (*M* = .14, *SD* = .22) opened a higher proportion of layers from an egocentric right-to-left fashion than those in the 0° condition (*M* = .03, *SD* = .12), *p* = 01, 95% CI[.03, .19]. There was no significant difference between the 90° condition (*M* = .08, *SD* = .22) and the 180° condition, *p* = 23, or the 0° condition, *p* = .15.

There was a main effect of age group, *F*(1, 88.91) = 13.91, *p* < .001, η_p_^2^ = .14. Children (*M* = .17, *SD* = .27) opened a higher proportion of layers from right-to-left than did adults (*M* = .03, *SD* = .12), 95% CI[.06, .20]. There was no main effect of trial, *p* = .75.

On the majority of trials, observers transformed their third-person viewpoint into allocentric coordinates, preferring to use the model’s precise actions. Nonetheless, children and face-to-face learners were more likely to use other coordinates when solving the puzzle box compared to other participants.

### Unnecessary actions

As a measure of overimitation, we examined the proportion of unnecessary model actions that participants performed on each trial and on each puzzle box layer.

#### By trial

There was a significant effect of trial, *F*(2, 174.80) = 9.00, *p* < .001, η_p_^2^ = .09. Participants performed more unnecessary actions on their first attempt at opening the puzzle box, on Trial 1 (*M* = .46, *SD* = .25), than on Trial 2 (*M* = .40, *SD* = .23), 95% CI[.02, .09], *p* = .005, or Trial 3 (*M* = .38, *SD* = .25), 95% CI[.04, .11], *p* < .001. There was no difference between unnecessary actions on Trial 2 and Trial 3, *p* = .17. There was no main effect of viewpoint, *p* = .67, or age group, *p* = .31.

#### By puzzle box layer

There was a significant effect of layer, *F*(3, 268.55) = 13.23, *p* < .001, η_p_^2^ = .13. Participants performed fewer unnecessary actions as they progressed through the layers of the puzzle box. There was no main effect of age group, *p* = .28, but there was an interaction between layer and age group, *F*(3, 268.55) = 3.00, *p* = .03, η_p_^2^ = .03. Adults (*M* = .58, *SD* = .35) performed more unnecessary actions on the first layer than children (*M* = .43, *SD* = .35), 95% CI[.008, .29], *p* = .04. There was no main effect of viewpoint, *p* = .68.

There were high levels of imitation of the model’s actions even when those actions had no functional utility. Overimitation was greatest on the first attempt and first layer of the box, consistent with a primacy effect. However, this was not influenced by viewpoint.

### Time to puzzle solution

Success in opening the puzzle box was near perfect in adults. To assess learning efficiency, we examined how quickly participants were able to open the puzzle box on each trial and each puzzle box layer. Five participants were excluded from trial analysis because they never completed Layer 4 and so failed to open the box.

#### By trial

There was a main effect of trial, *F*(2, 164.96) = 23.35, *p* < .001, η_p_^2^ = .22. Consistent with learning, time to open the puzzle box decreased linearly with repeated practice. Participants were faster on Trial 3 (*M* = 49.62_seconds_, *SD* = 26.54) than on Trial 1 (*M* = 67.25_seconds_, *SD* = 23.09), 95% CI[12.69, 23.20], p < .001, or Trial 2 (*M* = 56.37_seconds_, *SD* = 25.73), 95% CI[1.62, 12.00], *p* = .01. Participants were faster on Trial 2 than on Trial 1, 95% CI[5.96, 16.30], *p* < .001.

**Fig 5:**
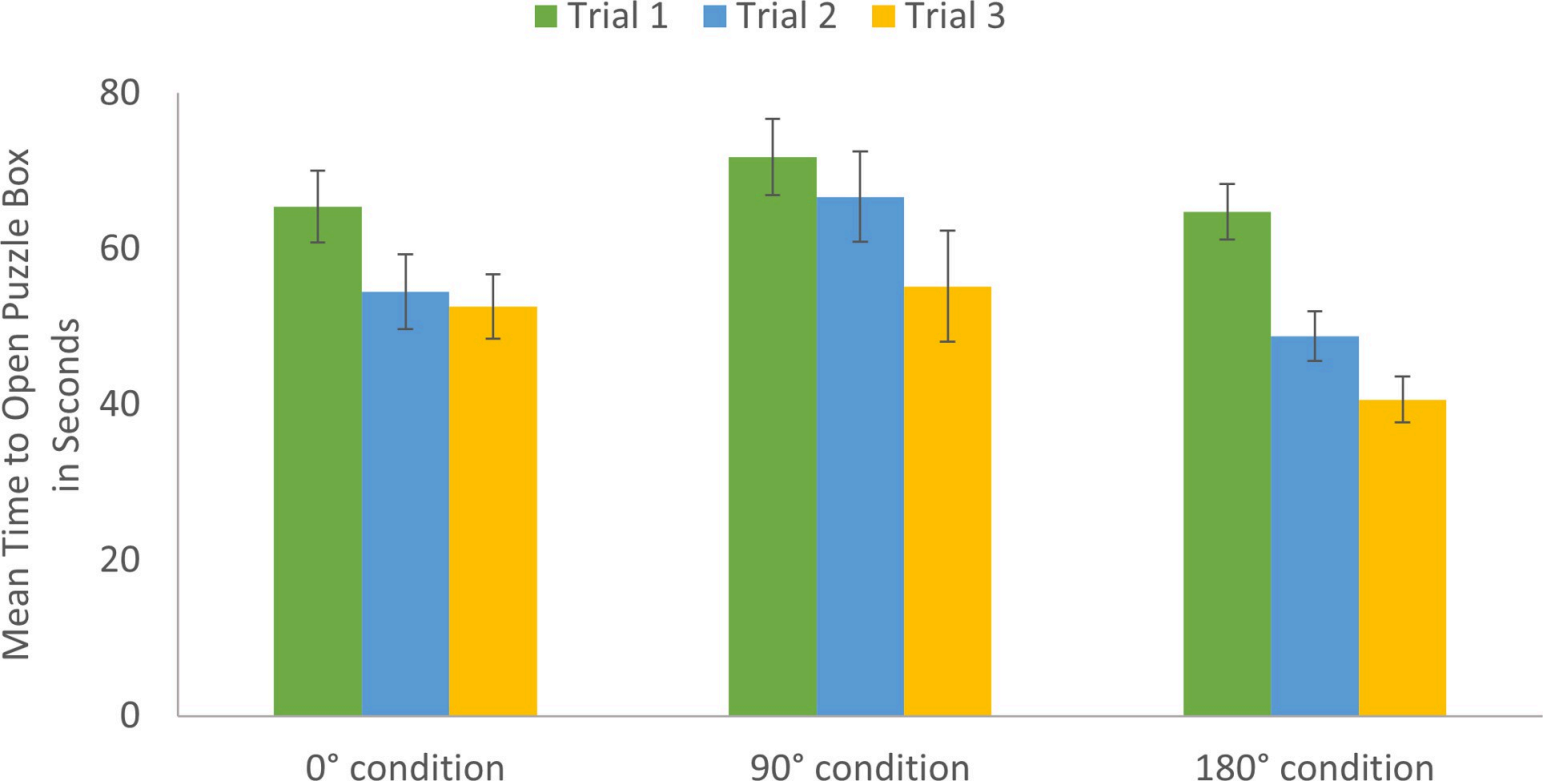
Mean time to open puzzle box by trial and viewpoint condition. Participants were significantly faster on the last trial than on the first or second trials and were significantly faster on the second trial than on the first trial. The 180° condition was significantly faster than the 90° condition, and the 0° condition was marginally faster than the 90° condition.

There was a main effect of viewpoint, *F*(2, 81.87) = 4.74, *p* = .01, η_p_^2^ = .10 (Fig 5). There was a curvilinear relation between viewpoint and time to open the puzzle box. The 180° condition (*M* = 51.80_seconds_, *SD* = 19.93) was significantly faster than the 90° condition (*M* = 64.68_seconds_, *SD* = 31.27), 95% CI[4.86, 23.23], *p* = .003. The 0° condition (*M* = 57.28_seconds_, *SD* = 24.85) was marginally faster than the 90° condition, 95% CI[0.13, 17.95], *p* = .05. There was no significant difference between the 0° condition and 180° condition, *p* = .26.

There was a main effect of age group *F*(1, 83.94) = 28.73, *p* < .001, η_p_^2^ = .25 (Fig 3B). Overall, adults (*M* = 50.63_seconds_, *SD* = 23.04) were faster than children (*M* = 72.41_seconds_, *SD* = 25.94), 95% CI[13.40, 29.20] at opening the puzzle box.

#### By puzzle box layer

There was a main effect of puzzle box layer, *F*(3, 263.81) = 154.29, *p* < .001, η_p_^2^ = .64. Time on each layer increased as participants progressed through the puzzle box, with the final layer representing the greatest challenge.

As expected from the trial analysis, there was a main effect of viewpoint, *F*(2, 86.99) = 3.59, *p* = .03, η_p_^2^ = .08. Collapsing across layers, the 180° condition (*M* = 9.73_seconds_, *SD* = 8.24) was significantly faster than the 90° condition (*M* = 15.89_seconds_, *SD* = 19.27), 95% CI[1.58, 10.62], *p* = .009. There was no significant difference between the 0° condition (*M* = 12.68_seconds_, *SD* = 13.20) and the 90° condition, *p* = .17, or the 180° condition, *p* = .18.

**Fig 6:**
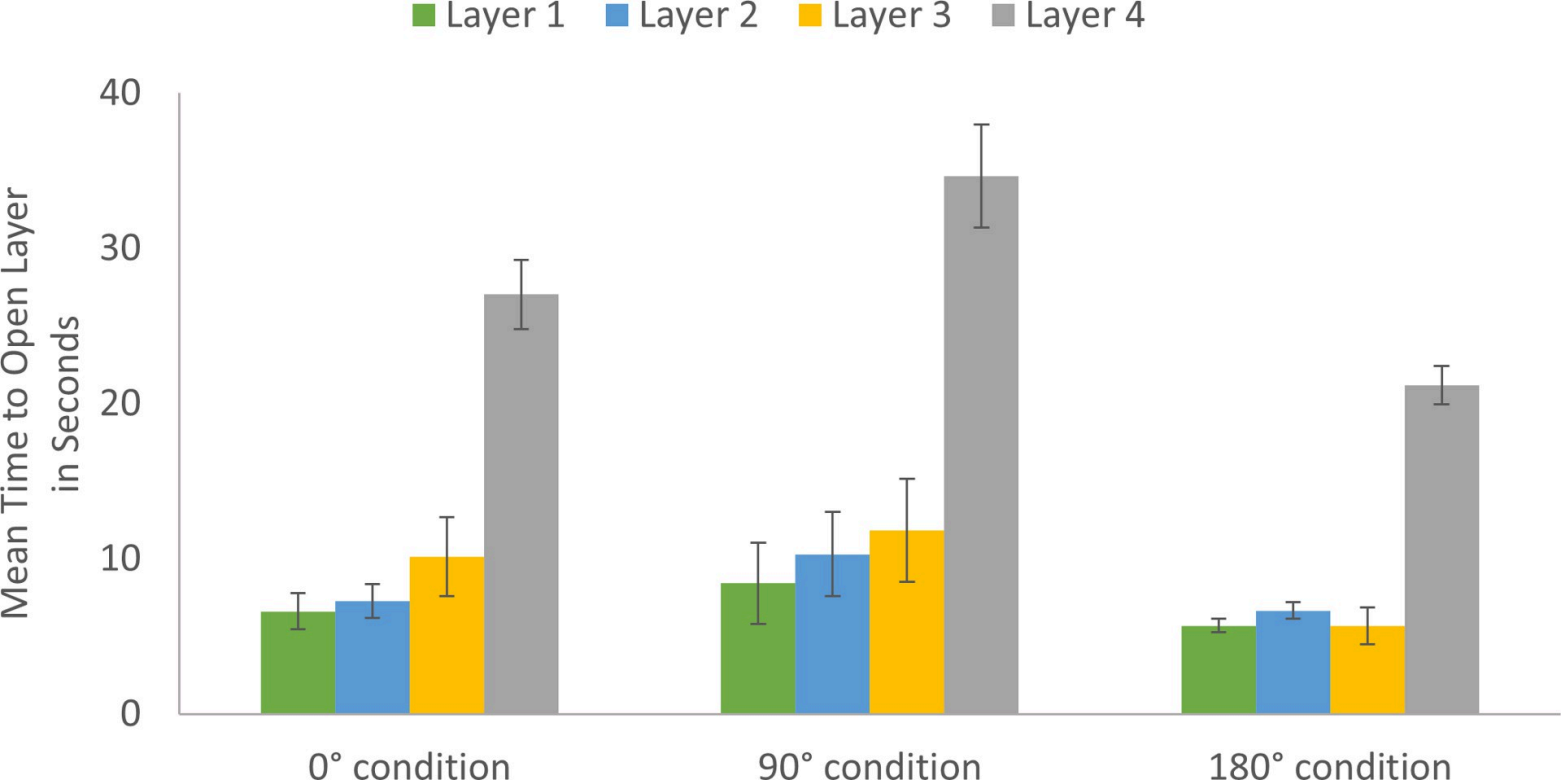
Mean time by puzzle box layer and viewpoint condition. On Layer 3, the 180° condition was marginally faster than the 90° condition. On Layer 4, the 180° condition was significantly faster than the 0° and 90° conditions, and the 0° condition was significantly faster than the 90° condition.

There was a significant interaction between layer and viewpoint, *F*(6, 263.83) = 3.48, *p* = .002, η_p_^2^ = .07 (Fig 6). The effect of viewpoint was most pronounced for the later layers of the puzzle box (Fig 6). Again, we found a curvilinear relationship between observational viewpoint and time. On Layer 3, the 180° condition (*M* = 5.69_seconds_, *SD* = 6.63) was marginally faster than the 90° condition (*M* = 11.83_seconds_, *SD* = 18.14), 95% CI[0.08, 11.25], *p* = .05. On the most challenging layer, Layer 4, the 180° condition (*M* = 21.21_seconds_, *SD* = 6.69) was significantly faster than both the 90° condition (*M* = 34.67_seconds_, *SD* = 17.62), 95% CI[7.61, 18.94], *p* < .001, and the 0° condition (*M* = 27.03_seconds_, *SD* = 12.41), 95% CI[0.48, 11.61], *p* = .03. On Layer 4, the 0° condition was faster than the 90° condition, 95% CI[1.67, 12.79], *p* = .01.

As with the trial analysis, there was a main effect of age group, *F*(1, 87.51) = 11.38, with adults (*M* = 10.37_seconds_, *SD* = 11.84) opening each layer more quickly than children (*M* = 16.77_seconds_, SD = 17.34), 95% CI[2.66, 10.30], p = .001, η_p_^2^ = .12.

Adults were faster than children in opening the puzzle box, but both became more proficient with practice. The effect of viewpoint was curvilinear and thus inconsistent with a mental rotation account. A 180° observational viewpoint resulted in the fastest puzzle box solving. Face-to-face observation promoted efficient puzzle solving, even when accounting for the number of unnecessary actions performed. More than first versus third-person observer-model reference frames, there was a special role for face-to-face learning that overcame disparity in visual perspectives to enhance puzzle solving. Further, the benefit of face-to-face learning was most prominent on the most challenging layer of the puzzle box.

#### Seat choice

There was evidence of a systematic seat choice preference. Overall, participants were more likely to choose the model’s seat (*n* = 36) than their observer’s seat (*n* = 21), *X*^2^(1, 61) = 6.45, *p* = .01, or the novel seat (*n* = 4), *X*^2^(1, 61) = 35.75, *p* < .001, and were more likely to choose the observer’s seat than the novel seat, *X*^2^(1, 61) = 12.88, *p* < .001.

**Fig 7:**
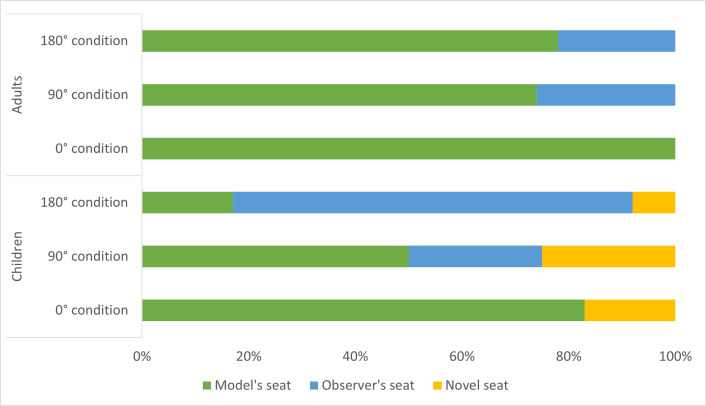
Percentage of participants choosing each seat by viewpoint condition. Adults in the 90° and 180° conditions chose the model’s seat significantly more often than the observer’s seat. Children in the 180° condition chose their observer’s seat significantly more often than the model’s seat.

To examine the other factors of interest, we conducted a binomial logistic regression that included viewpoint, age group, and AQ score in the model. As so few chose the novel seat (*n* = 4), we excluded these participants to simplify analysis to observer (egocentric) and model (allocentric) seat choice. There was a significant interaction between viewpoint and age group, *F*(1, 61) = 4.03, *p* = .04 (Fig 7). For adults in the third-person viewing conditions, both the 90° condition, *X*^2^(1, 19) = 6.74, *p* = .009, and 180° condition, *X*^2^(1, 18) = 9.00, *p* = .003, chose the model’s seat (i.e., changed seats to the model’s perspective) more often than their observation seat. In contrast, for children in the third-person viewing conditions, the 180° condition chose the observation seat more often than the model’s seat, *X*^2^(1, 11) = 6.55, *p* = .01, while children in the 90° condition were equally likely to choose the observation and the model’s seat, *p* = .35. Adults preferred to solve the puzzle box from an allocentric perspective, and thus a vantage point facilitating imitation of the model’s specific actions. Children preferred an egocentric perspective, suggesting their implicit viewpoint preference was not to embody the model and imitate the model’s specific actions.

## Discussion

Although visual (VPT) and social (SPT) perspective taking work in tandem to support social learning, each suggests a different relationship between the observation of knowledge and its social transmission. Visuospatial learning requires seeing the world from another’s perspective, which utilizes VPT. VPT depends upon mental rotation, so visuospatial learning should be most difficult from a 180° viewpoint, when learning face-to-face. Here we show that face-to-face learning can supersede the challenge of differences in visual perspective and the requirement for mental rotation. While a shared first-person visual perspective increased imitation (the learning of specific functional actions), replicating prior findings on action imitation [11], face-to-face learning enhanced goal emulation. Face-to-face learning also increased the likelihood of discovering a novel solution and, critically, resulted in more efficient/faster puzzle solving. The effect of the 180° observational viewpoint translated into improved complex visuospatial puzzle solving that persisted across trials.

Observational viewpoint had a large impact on imitation and learning to solve the puzzle box, but in opposite ways. Participants who engaged in face-to-face learning solved the puzzle box faster than those who sat next to or perpendicular to the model. Instead of a linear relationship between viewpoint and time, time to open the puzzle box increased between a 0° and 90° viewpoint but decreased between a 90° and 180° viewpoint. This was especially true on the most challenging final layer of the puzzle box, which required tool use. More than sharing a visual perspective, it appears that sharing a mental perspective supports the social transmission of knowledge.

In particular, face-to-face learning enhanced goal emulation over imitation. These related forms of social learning differ in how an outcome is achieved. Imitation achieves a model’s goal utilizing a model’s strategy, whereas goal emulation achieves the goal without utilizing the model’s strategy [14, 36]. The 180° condition was the most likely to discover a novel solution to the puzzle box and relied the least on the model’s solution. These participants also engaged in more motor mirroring. The 180° condition opened the puzzle box from right-to-left more often than participants in the 0° condition, suggesting an egocentric rather than allocentric perspective. This less faithful imitation was rewarded. In deviating from the model, participants achieved their goal of opening the puzzle box more quickly.

Overall, adults were more faithful imitators than children, exhibiting less goal-oriented behavior. Adults adopted the model’s solution more often than children and overimitated on the first layer more than children. In a sense, adults were more restricted learners than children. Adults focused on recreating the model’s actions rather than the end result. While this can be an effective way to develop expertise in an area, persistent imitation may hinder self-sufficiency and originality. This supports past findings that adults overimitate more than children, resulting in less efficient learning [37]. Additionally, children are more flexible learners than adults and engage in more exploration during learning [38].

We saw this same imitative behavior in adults’ seat choice. Overwhelmingly, adults chose the model’s seat when choosing where to sit. In contrast, children in the 180° condition chose the original seat from which they learned, further demonstrating a focus on goal achievement rather than model affiliation. This behavior was not due to egocentricity. If children were acting egocentrically, then all children should have returned to their original seat. Instead, children in the 90° condition were equally likely to choose the model’s seat as the observer’s seat. Perhaps strict imitation becomes ingrained through years of learning experience. Highly structured classroom activities cause declines in creativity [39], and classroom activities that encourage exploration promote creativity [40, 41]. As the number of years in formal educational settings increases, adults may become less divergent thinkers, depending less on self-generated learning and innovation.

There are a few limitations to our results and the conclusions we can draw from them. First, one possible explanation for these findings is that participants who were less imitative (i.e., the 180° condition, children versus adults) simply forgot the model’s actions or did not learn from observing the model. However, the 180° condition, which was the most imitative condition, was also the fastest condition. This suggests that non-imitation was not indicative of poor learning. Furthermore, if there was reduced learning/increased forgetting, then it was restricted to a diminished primacy effect (first trial, first layer of puzzle box). Participants’ memory for the unnecessary actions stabilized with practice; their performance of the model’s unnecessary actions did not significantly decrease between the last two trials. Additionally, adults and children performed the same number of unnecessary actions on the last three layers of the puzzle box, suggesting equivalent memory for these actions. Second, although a 180° observational viewpoint improved learning to solve the puzzle box in both adults and children, our results are not clear on whether the strength of this effect may change with age. It is interesting to note that face-to face learning caused adults to look more like child learners—less imitative and more exploratory. Future work is needed to systematically determine whether face-to-face learning is equally beneficial across development.

Lastly, we did not find an interaction between our control measure of SPT (normative autistic traits) and observational viewpoint. One explanation is that there was limited variability in our sample; the highest score among participants was below the cutoff for clinical levels of autistic traits. A second possibility is that higher autistic trait participants were better at mental rotation [42] and had enhanced spatial skills [43], so they were not affected by the increased demand for mental rotation at the 180° viewpoint. If high autistic traits are simultaneously related to better VPT and worse SPT, then this could lead to a null result. While we did not find them here, there is evidence of individual differences in personality that do modify observational learning [23].

Remarkably, the simple act of sitting across from someone can help overcome limitations in shared visual perspective. As suggested earlier, face-to-face learning may improve SPT through social affordances that enhance mind reading. Eye gaze, in particular, is a powerful pedagogical cue. Eye gaze promotes learning [19, 20], and children learn early on that social behaviors such as eye contact signal important information and carefully attend to these behaviors [44]. This may be why adults prefer to sit where a conversational partner is most visible [45, 46]. More than spatial proximity, face-to-face interaction may provide the foundation for social perspective taking, overcoming the structural constraint of different visuospatial perspectives.

In sum, we found that face-to-face learning overrode the inherent difficulty of taking another’s visual perspective. A 180° observational viewpoint enhanced goal emulation over action imitation and increased innovation during learning. The importance of observational viewpoint during learning has been undervalued. These insights can motivate research that considers the role of both visual and mental perspective during learning to enhance the balance between imitation and innovation.
